# Transcriptomic analysis supports collective endometrial cell migration in the pathogenesis of adenomyosis

**DOI:** 10.1016/j.rbmo.2022.05.007

**Published:** 2022-05-17

**Authors:** Junyu Zhai, Shang Li, Sushmita Sen, Júlia Vallvé-Juanico, Juan C. Irwin, Kim Chi Vo, Jipeng Wan, Yanzhi Du, Zi-Jiang Chen, Linda C. Giudice

**Affiliations:** 1 Center for Reproductive Sciences, Department of Obstetrics, Gynecology and Reproductive Sciences, University of California, San Francisco, San Francisco CA, USA; 2 Center for Reproductive Medicine, Ren Ji Hospital, School of Medicine, Shanghai Jiao Tong University Shanghai, China; 3 Shanghai Key Laboratory for Assisted Reproduction and Reproductive Genetics Shanghai, China; 4 Department of Obstetrics and Gynecology, Shandong Provincial Hospital, Cheeloo College of Medicine, Shandong University, Jinan Shandong, China; 5 Department of Obstetrics and Gynecology, Shandong Provincial Hospital Affiliated to Shandong First Medical University, Jinan Shandong, China; 6 National Research Center for Assisted Reproductive Technology and Reproductive Genetics, The Key Laboratory for Reproductive Endocrinology of Ministry of Education, Shandong Provincial Key Laboratory of Reproductive Medicine, Center for Reproductive Medicine, Shandong Provincial Hospital, Shandong University, Jinan, China

**Keywords:** Adenomyosis, Collective cell migration, ECM remodeling, GABA, Prolactin, RNA-seq

## Abstract

**Research question:**

Adenomyosis is a common uterine disorder of uncertain causes. Can transcriptomic analyses of the endometrium and myometrium reveal potential mechanisms underlying adenomyosis pathogenesis?

**Design:**

Transcriptomic profiles of eutopic endometrium and myometrium from women with and without diffuse adenomyosis and with symptomatic FIGO type 2–5 fibroids in the proliferative phase of the menstrual cycle were assessed using RNA sequencing and bioinformatic analysis. Differentially expressed genes (DEG) and potential pathways were validated by quantitative reverse transcription polymerase chain reaction, immunoblotting and Masson staining, using additional clinical samples.

**Results:**

Top biological processes in the endometrium of women with versus without adenomyosis, enriched from DEG, comprised inflammation, extracellular matrix (ECM) organization, collagen degradation and hyaluronan synthesis, which are key in cell migration and cell movement. Top biological processes enriched from DEG in the myometrium of women with versus without adenomyosis revealed ECM organization dysfunction, abnormal sensory pain perception and gamma aminobutyric acid (GABA) synaptic transmission. Dysregulation of prolactin signalling was also enriched in eutopic endometrium and in the myometrium of women with adenomyosis.

**Conclusions:**

Overall, our results support the invasive endometrium theory in the pathogenesis of adenomyosis, in which inflammation induces ECM remodelling resulting in a track for subsequent endometrial collective cell migration and onset of adenomyosis. Moreover, abnormal myometrial GABA synaptic transmission may contribute to dysmenorrhoea in women with adenomyosis and is a possible target for novel therapeutic development. Prolactin signalling abnormalities may serve as another opportunity for therapeutic intervention.

## INTRODUCTION

Adenomyosis is a common, hormonally driven uterine disorder occurring in 8–27% of reproductive age women (Kissler et al., 2008). It is associated with uterine enlargement, heavy menstrual bleeding (HMB), chronic pelvic pain, infertility and miscarriage (Harada et al., 2016). The pathognomonic feature of adenomyosis is the abnormal, heterotopic location of endometrial epithelial cells and stromal fibroblasts in the myometrium where they elicit hyperplasia and hypertrophy of surrounding smooth muscle cells (Zhai et al., 2020). The mechanisms and pathogenesis of how the adenomyotic lesions develop are uncertain, although the endometrial, myometrial compartments, or both, have been suggested as prime contributors.

One hypotheses involves enhanced invasion of the endometrial basalis through an injured or abnormal junctional zone into the myometrium (Zhai et al., 2020), via epithelial-to-mesenchymal transition (EMT) in early disease progression and collective cell migration in later invasion (Garcia-Solares et al., 2018). Junctional zone injury can be iatrogenic, e.g. caused by uterine surgery, or physiologic through microtissue injury and repair (TIAR) after each menstrual cycle (Leyendecker et al., 2009). Notably, adenomyosis lesions have been reported in the myometrium of women who lack functional endometrium, e.g. in those with Mayer–Rokitansky–Kuster–Hauser syndrome (Chun et al., 2013) or Asherman’s syndrome (Wang et al., 2021), and so other mechanisms may also be operational (Hoo et al., 2016).

An alternative hypothesis is the de-novo metaplasia theory (Garcia-Solares et al., 2018) wherein adenomyotic lesions derive from embryonic and adult stem cells in the myometrium *per se*. The stimuli for transformation of these cells are not clearly defined, and whether the myometrium is intrinsically abnormal in women with adenomyosis is yet to be determined.

Functional abnormalities believed to contribute to the pathogenesis of adenomyosis include increased endometrial cell proliferation, high invasive capacity of endometrial stromal cells, epithelial-to-mesenchymal transition and aberrant TIAR induced by microtrauma and trauma at the endometrial–myometrial interface (Benagiano et al., 2012; Zhai et al., 2020). Eutopic endometrium (lining the uterus) and ectopic endometrium of adenomyosis lesions in the myometrium aberrantly display activation of interleukin 6 (IL-6) and ERK/MAPK signalling, although studies are limited (Xiang et al., 2019). The myometrium also contributes to the pathogenesis and pathophysiology of adenomyosis, as increased uterine contractility, which is induced by overexpression of the oxytocin receptor in women with symptomatic adenomyosis, and is associated with dysmenorrhoea, common in this disorder (Guo et al., 2013). To date, transcriptomic analyses of the myometrium in the pathogenesis of adenomyosis are lacking.

The aim of the present study was to investigate potential mechanisms underlying the pathogenesis and pathophysiology of adenomyosis, with a focus on the endometrium and myometrium, and to potentially identify druggable targets to control its associated symptoms. To this end, endometrial and myometrial transcriptomic signatures and associated biologic processes and signalling pathways were pursued, with the use of RNA-sequencing, in a well-defined hormonal milieu of women with and without diffuse adenomyosis. These analyses, along with target validation studies, identified biological processes and regulatory networks that support endometrial and myometrial dysfunction in adenomyosis and the theory of collective endometrial cell migration in the pathogenesis of this disorder.

## MATERIALS AND METHODS

### Clinical samples

Endometrium and myometrium of women with adenomyosis and controls without adenomyosis were collected from hysterectomy specimens. Patients with adenomyosis were identified through clinical history and symptoms, and ultrasound, magnetic resonance imaging, or both. Histologic evaluation of hysterectomy specimens confirmed diagnosis, along with International Federation of Gynecology and Obstetrics (FIGO) type 2–5 uterine fibroids. Controls had undergone hysterectomy owing to symptomatic FIGO type 2–5 uterine fibroids, HMB, or both. Although it was not possible to collect myometrial tissue from normal controls, areas near uterine fibroids were avoided using immunohistochemistry (IHC) when selecting uterine tissue for RNA-sequencing, with the aim of minimize the effect of uterine fibroids on the transcriptome data. Full thickness uterine specimens (including endometrium and myometrium) were collected and stored at −80°C. Endometrium and myometrium were dissected from the frozen full thickness tissue using a surgical blade and away from areas of fibroids. All participants (*n* = 16 cases; *n* = 15 controls) were in the proliferative phase of the menstrual cycle, confirmed by endometrial histology (Noyes et al., 1975). Participant clinical characteristics are presented in Supplementary Table 1. All participants were documented as not pregnant and had not received hormonal or gonadotrophin releasing hormone agonist (GnRHa) treatments for at least 3 months before tissue sampling. Out of the 31 samples, six cases and five controls were used for RNA sequencing. The other 10 cases and 10 controls were used for validation using quantitative reverse transcription polymerase chain reaction (qRT-PCR). Of these, six cases and six controls were also used to validate protein using western blotting. The clinical samples were collected from the Human Endometrial Tissue and DNA Bank at the University of California, San Francisco, under an approved human subject’s protocol, which was initially approved in November 2010, with continuing review approval annually to date (IRB number 10–02786) and Ren Ji Hospital, School of Medicine, Shanghai Jiao Tong University (IRB number 2019122704) under an ongoing protocol, approved initially in 2019, after written informed consent was obtained from all participants.

### RNA extraction and sequencing

Total RNA was extracted from the endometrium and myometrium separately in six cases and five controls using the NuleoSpin RNA isolation Kit (Macherey-Nagel, Allentown, PA, USA). RNA quality was detected using a bioanalyzer; renewable identification numbers of all RNA samples were over 7. RNA sequencing library preparation was carried out as described previously (Klohonatz et al., 2019). Briefly, the Illumina TruSeq RNA Library Prep Kit (Illumina, San Diego, CA, USA) was used to prepare the mRNA sequencing library. The quality and concentration of all libraries were analysed with an Agilent Bioanalyzer (Agilent, Santa Clara, CA, USA). The Illumina Hiseq 2500 sequencing system (Illumina, San Diego, CA, USA) was used for mRNA sequencing, and 150–bp paired–end FASTQ read files were generated. The quality of fastq files was tested using the FastQC (Ward et al., 2020). A raw count of reads per gene was also obtained with STAR (Dobin et al., 2013). The data have been deposited in the NCBI GEO database (GSE190580). R/Bioconductor package (v1.20.0) was used to assess differential expression between cases and controls. Statistically significantly DEG were considered when *P* < 0.05 and log fold change was over 2.

### Enrichment analysis

DAVID Bioinformatics Resources 6.8 (https://david.ncifcrf.gov/) was used for clustering of DEG. Gene Ontology analysis was used to identify possible molecular functions and to visualize the potential biological translation of DEG. Kyoto Encyclopedia of Genes and Genomes (KEGG) was used to analyse the potential functions of these genes. The R package ‘clusterProfiler’ was used for Gene Ontology and KEGG pathway enrichment analyses.

### Protein–protein interaction and network analysis among differentially expressed genes

Interactions and K-means clustering among DEG of myometrium in adenomyosis versus controls were analysed using STRING (http://www.string-db.org/). Moreover, the protein–protein interaction (PPI) among DEG of endometrium were also identified using STRING.

### Masson’s trichrome stain

Human endometrial tissues were fixed with 4% paraformaldehyde solution for 24 h and embedded in paraffin. Then, the tissue was cut into 5-μm thick pieces and placed on glass slides, which were then baked at 60°C for 1 h, routinely dewaxed, rinsed and stained with Masson trichrome staining. Samples were imaged by microscopy (Zeiss, Axio Vert. A1) (Zeiss, Oberkochen, Germany).

### Real-time quantitative polymerase chain reaction

Total RNA from endometrium and myometrium tissues (10 cases and 10 controls) was extracted separately using an Animal Total RNA Isolation Kit (Foregene, Chengdu, China) and then reverse-transcribed into cDNA using PrimeScript RT Master Mix (Takara, Dalian, China) and BIO-RAD C1000 Touch Thermal Cycler. The mRNA expression of target genes was detected using real-time quantitative polymerase chain reaction. Results were analysed using the ΔΔCt method. The ratio of a target gene to β*-ACTIN* expression was calculated and reported as the target mRNA level, as in Wara-aswapati et al. (2007). The primer sequences of targeted genes are presented in Supplementary Table 2.

### Western blot

Total cellular proteins were isolated from human endometrium (six cases and six controls) tissues using ice-cold radioimmunoprecipitation assay lysis buffer (Cowbiotech, Beijing, China) containing a protease inhibitor cocktail (Roche, Basel, Switzerland) and a phosphatase inhibitor (Roche, Basel, Switzerland) for 30 min followed by centrifuging at 4°C and 13523 × g for 10 min and supernatant collection. The concentration of total protein was determined using a Pierce BCA Protein Assay Kit (Thermo Scientific, Massachusetts, USA) (Li et al., 2018). Samples of protein (35 μg) were separated on 10% sodium dodecyl sulfate gels in 80–120 V for 1.5 h and then wet transferred to a nitrocellulose membrane at 200 mA for 1.5–2 h. After blocking in 5% bovine serum albumin for 1 h, the nitrocellulose membrane was incubated with primary antibody dilution buffer (Beyotime, Shanghai, China) diluted antibody against matrix metalloproteinase 1 (MMP1) (1:1000) (Proteintech, Wuhan, China), MMP13 (1:1000) (Proteintech, Wuhan, China), collagen III (COLIII) (1:1000) (Proteintech, Wuhan, China), and COL1A1 (1:1000) (Cell Signaling Technology, Danvers, MA, USA) at 4°C overnight. The nitrocellulose membrane was then incubated with diluted matched peroxidase-conjugated secondary antibody for 1 h at room temperature. Membranes were then incubated with ECL Western blotting substrate (Merck Millipore, Billerica MA, USA) and immunoreactive bands visualized using GBOX (Syngene, Cambridge, UK), and the ratio of target protein to GAPDH (1:5000) (Proteintech, Wuhan, China) was calculated as previously reported (Li et al., 2018).

### Statistical analysis

Results are presented as mean ± SEM or SD. Differences between women with versus without adenomyosis were analysed in unpaired Student’s t-test with SPSS software (IBM, NY, USA). Statistical significance is shown as *P < 0.05, **P < 0.01, or ***P < 0.001.

## RESULTS

### Endometrium

#### RNA-sequencing

**FIGURE 1:**
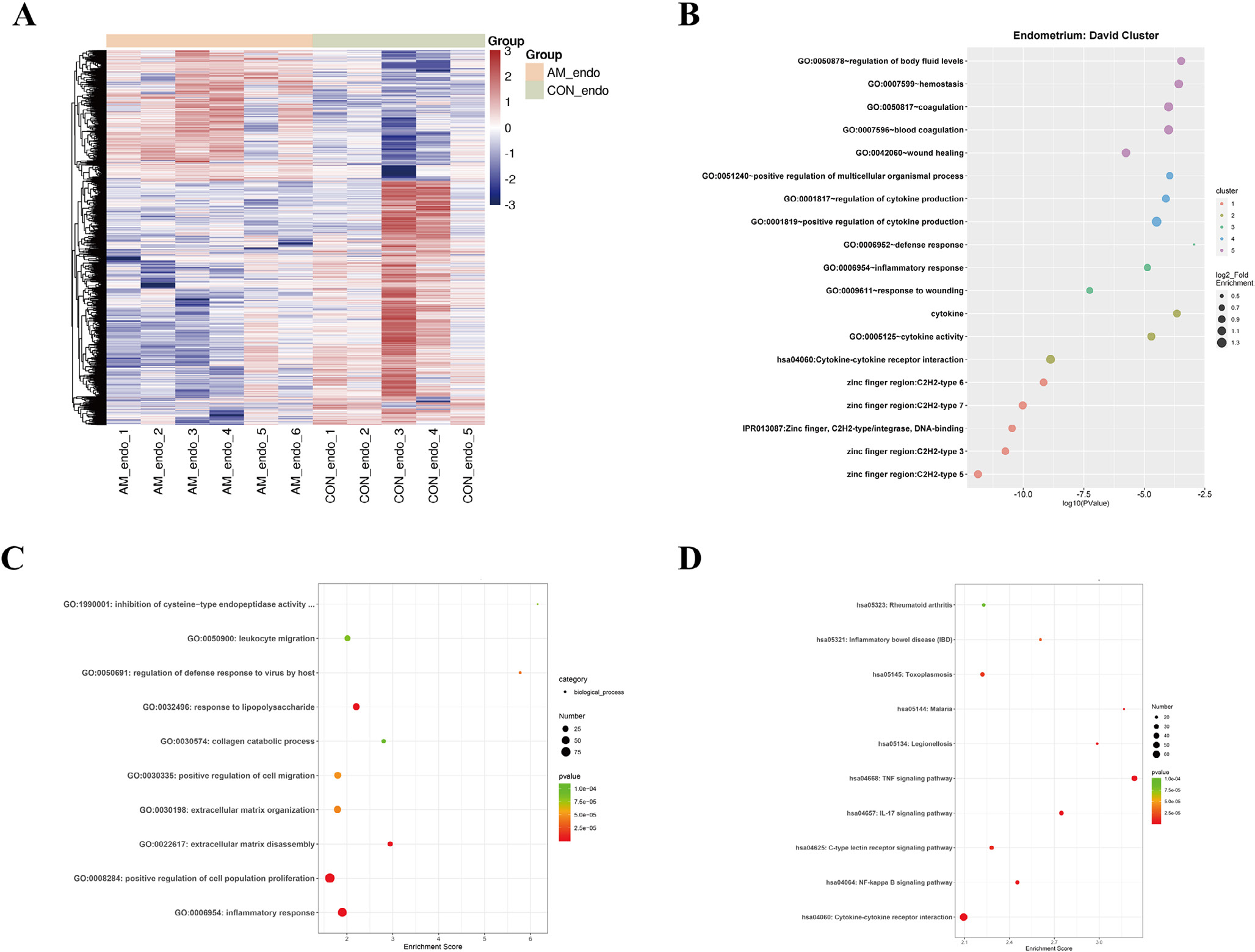
Expression and functional enrichment of differentially expressed genes (DEG) in the eutopic endometrium of women with and without adenomyosis. (A) Heatmap of DEG in the eutopic endometrium of women with versus without adenomyosis (cases = 6, controls = 5); (B) DAVID cluster of DEG in the eutopic endometrium of women with versus without adenomyosis; (C and D) Gene Ontology and Kyoto Encyclopedia of Genes and Genomes enrichments of DEG in the eutopic endometrium of women with versus without adenomyosis.

In the transcriptomic analysis of eutopic endometrium, 1014 DEG were identified in the comparison of six women with adenomyosis compared with five controls (FIGURE 1A). The clusters identified by DAVID from the DEG focused on wound healing, inflammatory response and DNA binding (FIGURE 1B). Similarly, Gene Ontology enrichment revealed dysfunction of inflammatory response, extracellular matrix disassembly and cell population proliferation in the endometrium of women with adenomyosis, whereas KEGG identified dysregulated inflammatory related signalling pathways (TNF, IL-17 and NF-kappa B signalling pathways) (FIGURE 1C and 1D).

#### Network analysis

**FIGURE 2:**
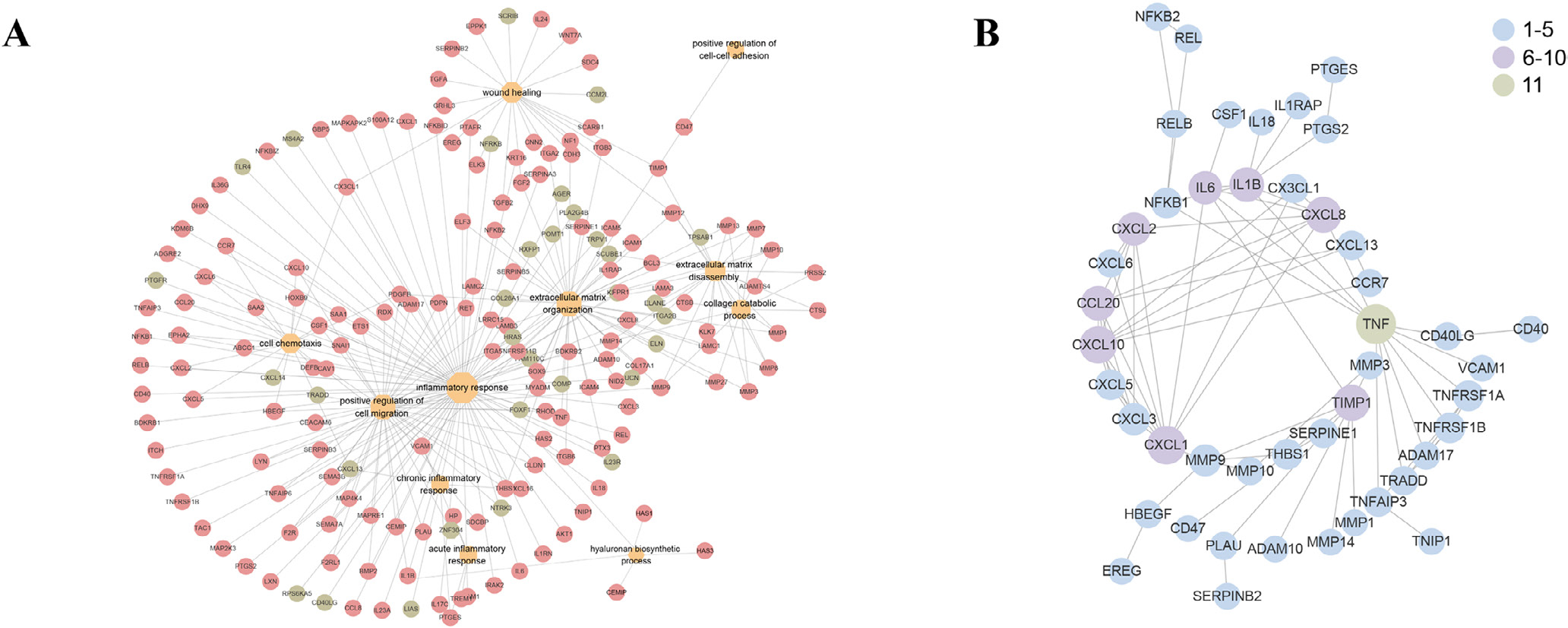
Target gene network and protein–protein interaction analysis among the differentially expressed genes (DEG) of eutopic endometrium of women with versus without adenomyosis. (A) Network of DEG belonging to the top enriched biological processes. Red: the upregulated DEG in the eutopic endometrium of women with versus without adenomyosis. Green: the downregulated DEG in the eutopic endometrium of women with versus without adenomyosis; (B) protein–protein interaction analysis among the DEG belonging to the top enriched biological processes that function in the endometrial dysfunction of women with adenomyosis. Blue: the gene is associated with one to five DEG. Purple: the gene is associated with six to 10 DEG. Green: the gene is associated with 11 DEG.

Protein–protein interaction and network analysis were conducted to investigate the interaction between inflammation and ECM remodelling and identify key molecules in these biological processes. Inflammatory response shares numerous DEG with ECM remodelling (ECM organization, ECM disassembly, collagen catabolic process and hyaluronan biosynthetic process), as well as positive regulation of cell migration in the analysis of endometrium of women with versus without adenomyosis (FIGURE 2A), indicating the close interactions between inflammatory response and ECM remodelling. Moreover, PPI analysis revealed that inflammatory factors, especially tumour necrosis factor (*TNF*), interleukin 1 beta (*IL1B*) and chemokines, correlated with many genes in ECM degradation and cell migration, including matrix metalloproteinase (*MMPs*) and a disintegrin and metalloproteases (*ADAMs*) (FIGURE 2B). Therefore, inflammatory induced ECM remodelling and subsequent activated cell migration in eutopic endometrium of women with adenomyosis is supported from the above analysis.

Several prominent biological processes were identified in the endometrium of women with versus without adenomyosis, including cell–cell adhesion and coupling, forming a migration track or ECM remodelling and driving force of cell migration (TABLE 1). Persistent cell adhesion and abnormal expression of chemokine (CXC motif) ligand/receptor (CXCL/CXCR) signalling pathways, essential in collective cell invasion (Strieter et al., 2006), were also detected in the endometrium of women with adenomyosis (TABLE 1). Therefore, these processes involved in creating a track for cell migration and persistent cell adhesion support a role for collective endometrial cell migration, driven by CXCL/CXCR signalling, in the development of adenomyosis.

#### Validation

**FIGURE 3:**
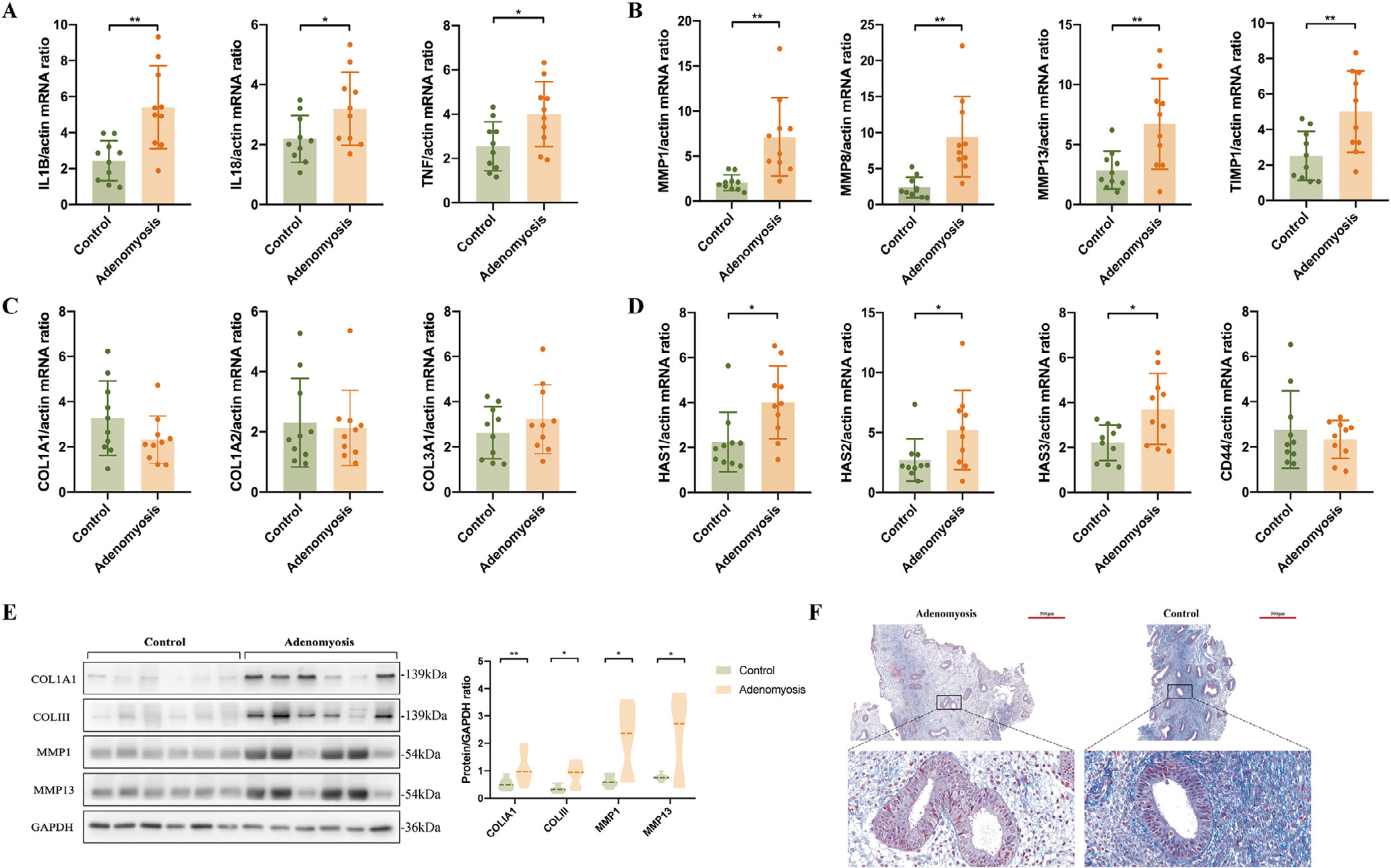
Validation of potential target genes in top enriched biological processes in the eutopic endometrium of women with versus without adenomyosis. (A) mRNA levels of *IL1B*, *IL18* and *TNF* in eutopic endometrium of adenomyosis patients (*n* = 10) and controls (*n* = 10) detected by quantitative reverse transcription polymerase chain reaction analysis (qRT-PCR). All endometrial samples were in the proliferative phase. β*-ACTIN* was used as reference gene for qRT-PCR analysis. *P*-values are 0.002, 0.041 and 0.022, respectively; (B) mRNA levels of *MMP1*, *MMP8*, *MMP13* and *TIMP1* in eutopic endometrium of adenomyosis patients (*n* = 10) and controls (*n* = 10) detected by qRT-PCR analysis. *P*-values are 0.002, 0.001, 0.008 and 0.0085, respectively; (C) mRNA levels of *COL1A1*, *COL1A2* and *COL3A1* in eutopic endometrium of adenomyosis patients (n = 10) and controls (n = 10) detected by qRT-PCR analysis; (D) mRNA levels of *HAS1*, *HAS2*, *HAS3* and *CD44* in eutopic endometrium of adenomyosis patients (*n* = 10) and controls (*n* = 10) detected by qRT-PCR analysis. *P*-values are 0.016, 0.049, 0.015 and 0.484, respectively. Data are presented as means ± SEM. *P < 0.05, **P < 0.01; (E) protein levels of COL1A1, COLIII, MMP1 and MMP13 in the endometrium of adenomyosis patients and controls. *n* = 6 for each group. GAPDH was used as reference control, and the ratio of band intensity of a target protein to that of the intensity of GAPDH was obtained at each target protein level (Li et al., 2018). The *P*-value of the protein level of COL1A1, COLIII, MMP1 and MMP13 in adenomyosis versus controls are 0.009, 0.024, 0.018 and 0.031, respectively. *P < 0.05, **P < 0.01; (F) Masson stain of eutopic endometrium in adenomyosis patients versus controls.

Key DEG of top enriched biological processes, especially inflammation and ECM remodelling, in the endometrium of women with adenomyosis were validated at the RNA and protein levels (FIGURE 3). *IL1B*, *IL18* and *TNF* were all significantly increased in women with adenomyosis compared with controls (*P* = 0.002, *P* = 0.041 and *P* = 0.022, respectively) (FIGURE 3A). Moreover, the enzymes responsible for collagen degradation, *MMP1*, *MMP8* and *MMP13*, and their natural inhibitor, tissue inhibitor of metallopeptidase 1 (*TIMP1*), also increased in the endometrium of women with adenomyosis (*P* = 0.002, *P* = 0.001, *P* = 0.008 and *P* = 0.0085, respectively), although changes in *COL1A1*, *COL1A2* and *COL3A1* mRNAs were not observed (FIGURE 3B and 3C). Hyaluronan is another critical component of the ECM, and the enzymes for hyaluronan synthesis, hyaluronan synthase *HAS1*, *HAS2* and *HAS3*, were also highly expressed in the endometrium of women with adenomyosis (*P* = 0.016, *P* = 0.049 and *P* = 0.015, respectively) without changes in their receptor, *CD44*, a cell-surface glycoprotein involved in cell–cell interactions, cell adhesion and migration (FIGURE 3D). To verify key proteins involved in ECM remodelling and collagen catabolic process, western blot was carried out. Increased MMP1 and MMP13 were detected in the endometrium of women with versus without adenomyosis (*P* = 0.018 and *P* = 0.031) (FIGURE 3E). COL1A1 and COLIII protein immunoreactivity was significantly increased (*P* = 0.009 and *P* = 0.024) in the absence of significant changes in the corresponding mRNA in endometrium of women with adenomyosis (FIGURE 3E and 3F).

### Myometrium

#### RNA-sequencing

**FIGURE 4:**
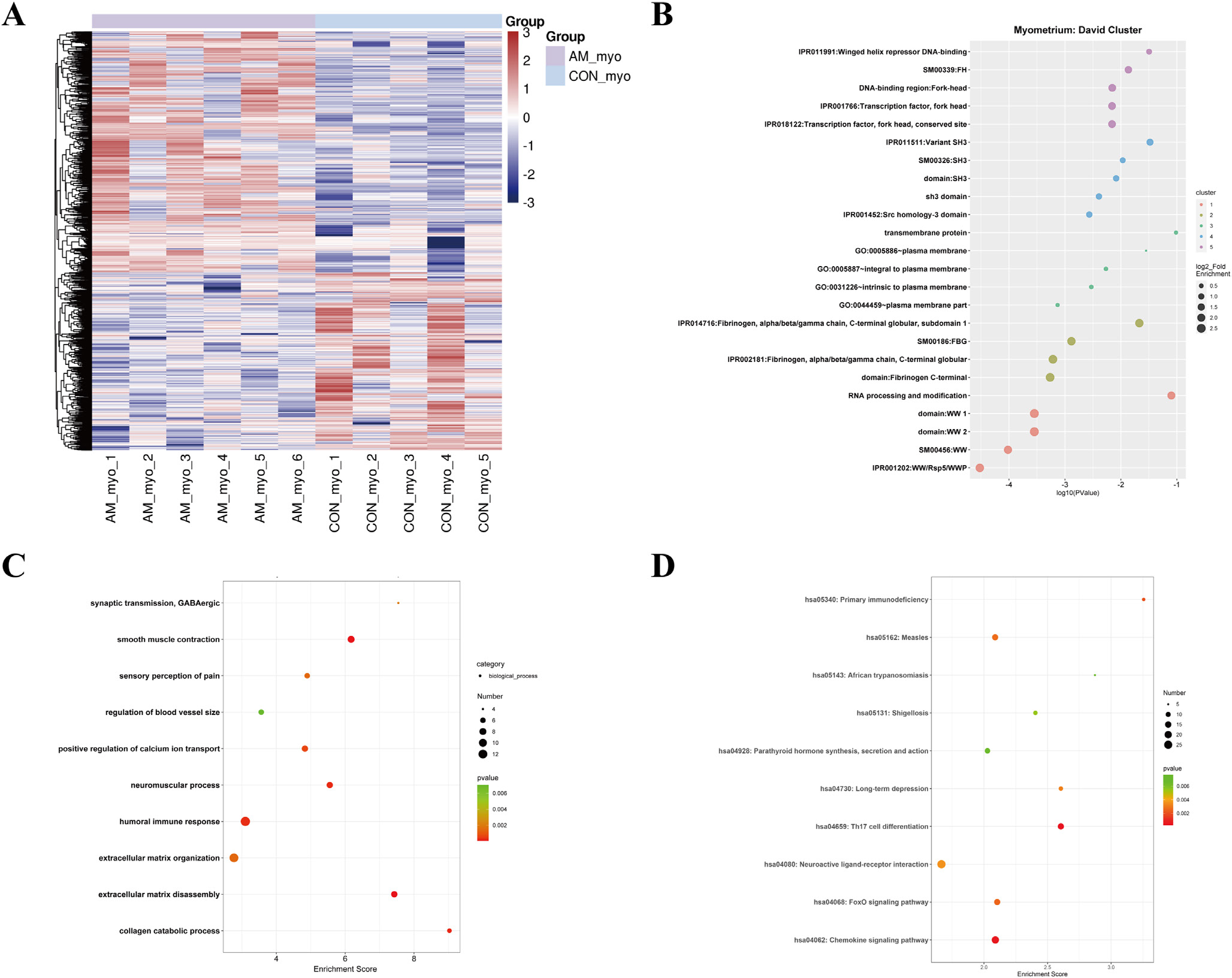
Expression and functional enrichment of differentially expressed genes (DEG) in the myometrium of women with versus without adenomyosis. (A) Heatmap of DEG in the myometrium of women with and without adenomyosis (*n* = 6 cases; *n* = 5 controls); (B) DAVID cluster of DEG in the myometrium of women with versus without adenomyosis; (C and D) Gene Ontology and Kyoto Encyclopedia of Genes and Genomes enrichments of DEG in the myometrium of women with versus without adenomyosis.

Myometrial dysfunction may also contribute to the pathogenesis of adenomyosis. In the present study, 1906 DEG were identified in the transcriptomic analysis of the myometrium of women with versus without adenomyosis (FIGURE 4A). Further enrichment analysis found that the myometrial layer of adenomyosis patients also presented ECM organization and collagen catabolic processes dysfunction, similar to endometrium (FIGURE 4B–4D). Interestingly, abnormalities were found in sensory pain perception as well as gamma aminobutyric acid (GABA) synaptic transmission in the myometrium of women with adenomyosis (FIGURE 4C), suggesting a neuropathic nature for chronic pelvic pain and dysmenorrhoea associated with this disorder.

#### Network analysis

**FIGURE 5:**
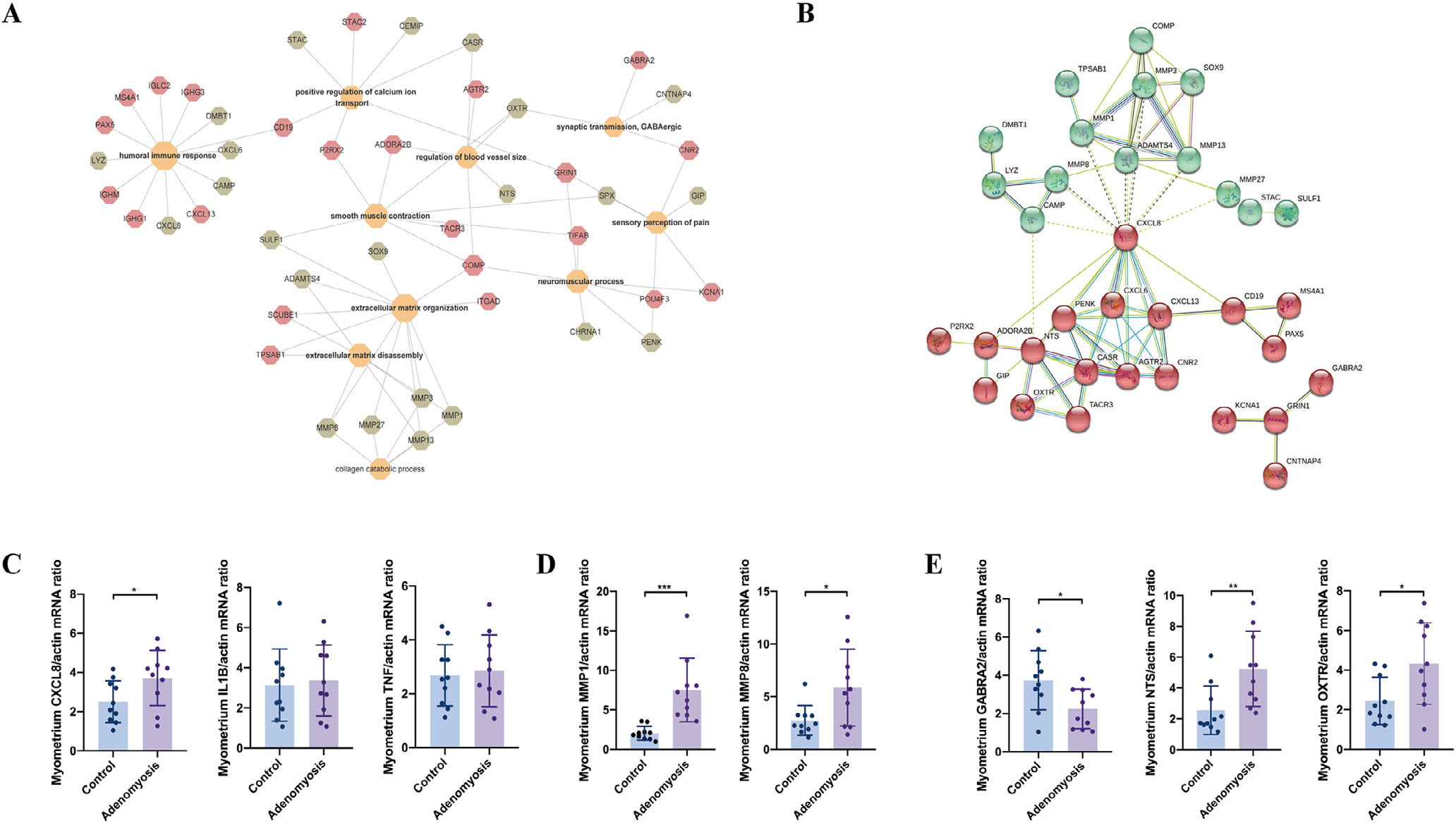
Target gene network analysis and validation in the myometrium of women with versus without adenomyosis. (A) Network of differentially expressed genes (DEG) belonging to the top enriched biological processes in the myometrium of adenomyosis patients versus controls. Red: upregulated DEG in the myometrium of women with and without adenomyosis. Green: downregulated DEG in the myometrium of women with versus without adenomyosis; (B) K-means clustering of DEG belonging to the top enriched biological processes using STRING. Red and Green indicate two different clusters; (C) mRNA levels of *CXCL8*, *IL1B* and *TNF* in the myometrium of adenomyosis patients (*n* = 10) and controls (*n* = 10) detected by quantitative reverse transcription polymerase chain reaction analysis (RT-qPCR) analysis. β*-ACTIN* was used as a reference gene for qRT-PCR analysis. *P*-values are 0.044, 0.769 and 0.760, respectively; (D) mRNA levels of *MMP1* and *MMP8* in the myometrium of adenomyosis patients (*n* = 10) and controls (*n* = 10) detected by qRT-PCR analysis. *P*-values are 0.0005 and 0.021, respectively; (E) mRNA levels of *GABRA2*, *NTS* and *OXTR* in the myometrium of adenomyosis patients (*n* = 10) and controls (*n* = 10) detected by qRT-PCR analysis. *P*-values are 0.020, 0.009 and 0.022, respectively. Data are presented as means ± SEM. *P < 0.05, **P < 0.01, ***P < 0.001.

From the network analysis, ECM remodelling and myometrial neural disorder interact with each other closely (FIGURE 5A). Moreover, K-means clustering of DEG involved in the top enriched Gene Ontology terms identified the association and key molecules that function in the dysregulated biological processes in endometrium of women with adenomyosis (FIGURE 5B). The analysis showed that ECM remodelling is mainly attributed to the green cluster, whereas neuropathic processes and humoral immune response belong to the red cluster (FIGURE 5B). Also, *CXCL8* may function as a mediator of both ECM remodelling and neuropathic dysfunction owing to its extensive contact with the DEG in the green and red clusters (FIGURE 5B).

#### Validation

In addition, mRNA levels of *CXCL8* were also significantly increased in the myometrium of women with adenomyosis compared with controls (*P* = 0.044) (FIGURE 5C) without significant changes in either *IL1B* or *TNF.* On the contrary, *MMP1* and *MMP8* mRNAs were significantly increased in the myometrium of women with adenomyosis (*P* = 0.0005 and 0.021, respectively) (FIGURE 5D), similar to that observed in the endometrium of women with disease (FIGURE 3B). For the neuropathic processes, synaptic transmission related genes were validated, including decreased gamma-aminobutyric acid type A receptor subunit alpha2 (*GABRA2)*, increased neurotensin (*NTS*) and oxytocin receptor (*OXTR)*, in the myometrium of women with adenomyosis versus without (*P* = 0.020, *P* = 0.009 and *P* = 0.022, respectively) (FIGURE 5E).

### Endometrium and myometrium

**FIGURE 6:**
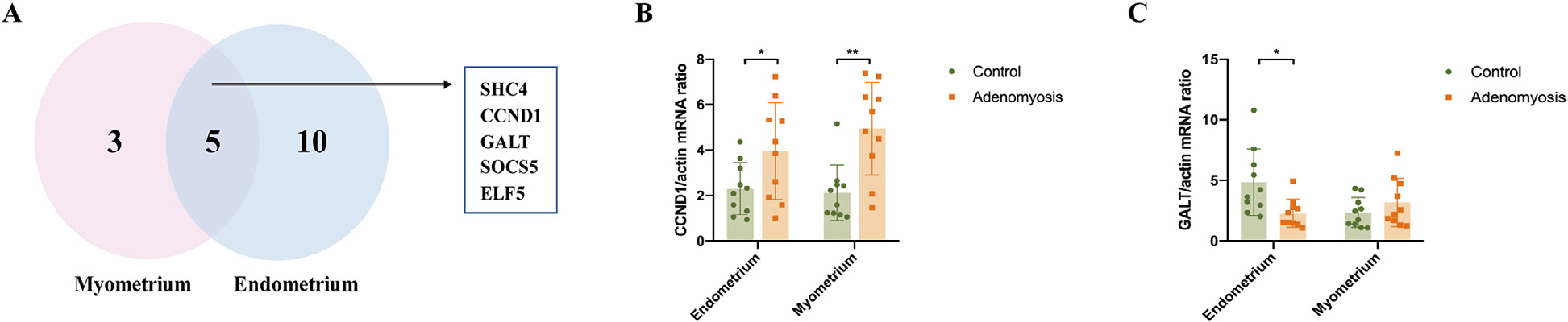
Potential target genes of prolactin (PRL) signalling pathways contributing to the dysfunction of both eutopic endometrium and myometrium in women with adenomyosis. (A) Venn diagram of the differentially expressed genes (DEG) enriched to PRL signalling pathways in eutopic endometrium and myometrium. Five common target genes were identified, which consist of *SHC4*, *CCND1*, *GALT*, *SOCS5* and *ELF5*; (B) the expression of *CCND1* mRNA in the eutopic endometrium (*P* = 0.044) and myometrium (*P* = 0.002) of women with and without adenomyosis (*n* = 10 for each group); (C) the expression of *GALT* mRNA in the eutopic endometrium (*P* = 0.014) and myometrium (*P* = 0.282) of women with and without adenomyosis (*n* = 10 for each group). *P < 0.05, **P < 0.01.

In the present analysis, 115 DGE commonly dysregulated in both the endometrium and myometrium of women with versus without adenomyosis were identified. The top enriched Gene Ontology terms are collagen catabolic process, immune response and chemotaxis (TABLE 2). Comparison of genes and pathways commonly dysregulated in both endometrium and myometrium of women with versus without adenomyosis revealed a role for prolactin (PRL) signalling, supporting a longstanding hypotheses for involvement of PRL and PRLR in the pathogenesis and pathophysiology of this disorder (Mori et al., 1981). In the present study, the PRL signalling pathway was enriched in DEG of both endometrium and myometrium of women with versus without adenomyosis (enrichment scores of 1.955 and 2.23, respectively). Five common DEG genes included *SHC4*, *CCND1*, *GALT*, *SOCS5* and *ELF5* (FIGURE 6A). In the present validation, *CCND1* mRNA was significantly increased in both the eutopic endometrium (*P* = 0.044) and myometrium (*P* = 0.002) of women with adenomyosis (FIGURE 6B), consistent with a role for *CCND1* in endometrial cell proliferation in women with adenomyosis. In addition, *GALT* mRNA expression was decreased in the eutopic endometrium (*P* = 0.014) of adenomyosis versus controls without significant changes in the myometrium (*P* = 0.282) (FIGURE 6C).

## DISCUSSION

### Insights into pathogenesis: collective endometrial cell migration theory

In the endometrium of women with versus without adenomyosis, biological processes and functional analyses derived herein revealed inflammation-induced ECM remodelling and cell cohesion and coupling, forming a migration track and driving force for guided cell migration. Previous studies have demonstrated a role for inflammatory factors in promoting ECM remodelling and subsequent cell migration in tumours (Lee and Heur 2013; Wang et al., 2017), and the observations herein provide supporting molecular evidence for this phenomenon in the endometrium of women with adenomyosis using whole genome transcriptomics. A recent publication on single cell RNA sequencing of endometrium from a woman with adenomyosis versus women with uterine fibroids is consistent with our findings (Liu et al., 2021).

Cell movement ranges from uncoordinated ruffling of cell boundaries to migration of single cells to collective motions of cohesive cell groups (Thuroff et al., 2019). Cell migration, the basis of cell invasion, comprises migration of single cells to position themselves in tissues and collective migration wherein cells remain connected as they move, resulting in migrating cohorts (Friedl and Gilmour, 2009). For the latter, cells remain physically and functionally connected during movement; multicellular polarity and ‘supracellular’ organization of the actin cytoskeleton generate traction and protrusion force for migration. Also, moving cell groups structurally modify the tissue along the migration path, either by clearing the track or by causing secondary ECM modification (Friedl et al., 2004; Montell, 2008). Previous studies have suggested the potential role of collective cell migration in the invasion process of deep endometriotic lesions and latter phases of adenomyosis (Donnez et al., 2015; Garcia-Solares et al., 2018). No direct evidence to date, however, has clearly demonstrated collective cell migration in adenomyosis development. In our data, however, the striking triad of cell cohesion and coupling form a migration track and guided cell migration among the biological processes derived from DEG in the endometrium of women with versus without adenomyosis. This supports endometrial collective cell migration into the myometrium, resulting in the development of ectopic endometrium lesions in the myometrium of women with adenomyosis. Whether collective cell migration plays a role in the onset of adenomyosis needs to be further verified through IHC of E-cadherin, β-catenin, N-cadherin and other biomarkers and in animal models.

Increased ECM organization and collagen catabolic process were detected in the endometrium and myometrium of adenomyosis cases with unclear pathogenesis. One of the most common causes is chronic injury and inflammation caused by hyperperistalsis of the junctional zone, which further leads to abundant myofibroblasts and collagen hyperplasia. Collective cell behaviour in response to mechanical injury is central to various regenerative and pathological processes (Jiang et al., 2020). Therefore, the trigger for this may be micro TIAR caused by hyperperistalsis (Leyendecker et al., 2009) or an iatrogenically injured endometrial–myometrial interface (junctional zone), involving local oestrogen signalling, inflammation and wound repair mechanisms. Importantly, women who have had a caesarean section or dilatation and curettage procedures have higher risk of developing adenomyosis (Upson and Missmer, 2020), consistent with this hypothesis.

Regarding mechanisms underlying collective cell migration from the endometrium to the myometrium leading to adenomyosis, the present data support a role for CXCL/CXCLR signalling, as direction of migration along a track depends on the polarity of cell clusters and chemokines within the anatomic niche (Zhou et al., 2010). What regulates dysfunctionality of endometrial CXCL/CXCLR signalling, the cell types and specific ligand/receptor pairs involved, and specific roles for this signalling family in the pathogenesis of adenomyosis, warrant further investigation. Notably, as collective cell movement is relevant for processes in morphogenesis, tissue repair and cancer invasion and metastasis, conserved mechanisms may be operational, as suggested by Garcia-Solares et al. (2018).

### Endometrium and myometrium: prolactin and adenomyosis

A possible role for PRL in adenomyosis was derived initially from an experiment conducted over 40 years ago wherein hypophyseal transplantation into mice uteri induced adenomyosis (Mori et al., 1981). Subsequently, infusion of PRL or administration of dopamine agonist causing hyperprolactinemia resulted in adenomyosis in the mouse (Singtripop et al., 1991). More recently, higher serum levels of PRL in women with adenomyosis compared with those without disease have been reported (Sengupta et al., 2013). In the present study, several members of the PRL signalling pathway (FIGURE 6) that are dysregulated in the endometrium of women with adenomyosis and are involved in cell proliferation, cell cycle progression and gluconeogenesis. For example, SHC4 is involved in PRL signalling and plays a role in cell proliferation, differentiation and survival (Ahmed and Prigent, 2017). These are important processes in the pathogenesis of adenomyosis. In endometrial cancer cells, autocrine PRL expression stimulates cell proliferation, migration and invasion, and promotes tumour growth, local invasion and metastases, processes that are important in adenomyosis pathogenesis.

Additionally, over-expression of PRL in the Ishikawa endometrial adenocarcinoma cell line increases cyclin D1 (*CCND1*) mRNA levels and enhances cell cycle progression (Ding et al., 2017). CCND1 is a key component of PRL signalling and may be a factor in endometrial cell proliferation and adenomyosis. Galactose-1-phosphate uridyl transferase (GALT) is a key enzyme in gluconeogenesis, which is inhibited by PRL/PRLRs via Foxo3a (Devi et al., 2009). Recently it was found to be associated with adenomyosis (Goumenou et al., 2000). *SOCS5*, another common DEG identified in PRL signalling, is a member of the suppressor of cytokine signalling (SOCS) protein family with controversial tumour-promoting and tumour-suppressive roles in cancer. Zhang et al. (2019a) reported that *SOCS5* overexpression promoted hepatic cancer cell migration and invasion *in vitro* by inactivating PI3K/Akt/mTOR-mediated autophagy. E74-like factor 5 (ELF5) also plays a key role in the processes of cell differentiation and apoptosis, whereas overexpression of *ELF5* inhibits migration and invasion of ovarian cancer cells (Zhang et al., 2019b). Previously published studies have shown that several additional factors can affect the PRL pathway. For example, nuclear receptor (NR) 4A modulates decidualization of endometrium by upregulating PRL via forkhead box O (FOXOA1) (Jiang et al., 2016). Notably, we found no changes in the expression of NR4A or FOXOA1 in our data, and this warrants further investigation. Overall, combining the published research with our results, local PRL signalling may contribute to dysfunction of endometrium and myometrium in women with adenomyosis via *SHC4*, *CCND1*, *GALT, SOCS5* and *ELF5*, with specific mechanisms awaiting further study.

### Myometrium: pain pathways and heavy menstrual bleeding

In the present study, myometrial transcriptomic analysis revealed a possible neuropathic nature of dysmenorrhoea in women with adenomyosis. Dysmenorrhoea is a clinical hallmark of adenomyosis (Upson and Missmer, 2020). It has been postulated that myometrial hypercontractility, caused by high expression of oxytocin receptors and increased contractile amplitude of uterine smooth muscle cells in the myometrium of women with versus without adenomyosis, are responsible for the severe dysmenorrhoea associated with the disease (Nie et al., 2010). Inflammatory factors, such as IL-1β and corticotropin releasing hormone, play a role in pain associated with deep infiltrating endometriosis (Carrarelli et al., 2016), a disorder that is physiologically and histologically similar to adenomyosis. Accumulating data indicate that sensory nerve-derived neuropeptides, such as calcitonin gene related-protein (CGRP), can accelerate the progression of endometriosis via their respective receptors, whereas adrenergic β2 receptor (ADRB2) agonists also are involved in facilitating lesion progression. More remarkably, lesional expression of ADRB2 correlated positively with the severity of dysmenorrheoa in women with endometriosis (Yan et al., 2019). Therefore, complex mechanisms, including mechanical movement, inflammatory factors and neuropeptides, likely play important regulatory roles in dysmenorrhoea in adenomyosis. In the present study, abnormal expression of spexin (*SPX*), cannabinoid receptor 2 (*CNR2*) and POU class 4 homeobox (*POU4F3*) may have been involved in sensory perception of pain in the myometrium of women with and without adenomyosis, relevant to dysmenorrhoea (FIGURE 5A). GABA, a neurotransmitter involved in pain sensation, functions as an inhibitory synaptic transmitter (Yam et al., 2018). GABRA2 is a member of the GABAA receptor family that signals inhibitory functions of GABA in the central nervous system and in peripheral tissues, including rat and human uterine myometrium and in smooth muscle vasculature of the endometrium (Human Protein Atlas) (Greenfield et al., 2002). The neurosteroid allopregnanolone binding to GABAAR has been proposed to inhibit myometrial contractility, involving the π subunit (Greenfield et al., 2002). Dysregulation of GABA synaptic transmission in our in-silico analysis of myometrium from women with adenomyosis supports a local neuropathic disorder in adenomyosis myometrium, likely involving enhanced myometrial contractility and pain, in addition to GABA’s role in the central nervous system. Decreased expression of *GABRA2* in the myometrium of adenomyosis women and further definition of the different subunits that confer tissue-specific expression may provide potential targets for drug development and underpin future mechanistic studies aimed to minimize pain associated with adenomyosis (Vannuccini et al., 2017).

Heavy menstrual bleeding is a common symptom in patients with adenomyosis. Previous studies have indicated that mechanisms underlying HMB in adenomyosis involve neoangiogenesis, abnormal uterine contractility and high microvessel density (Harmsen et al., 2019). Events leading to increased proangiogenic factor expression, such as vascular endothelial growth factor, are triggered by TIAR, hypoxia and hormonal dysfunction. Therefore, HMB may result from both endometrial and myometrial pathology in women with adenomyosis. In the present study, most participants with adenomyosis and only three controls had HMB. The transcriptomic result highlighted the increased OXTR in myometrium and dysregulated ECM changes, collagen degradation and inflammation in the endometrium of women with adenomyosis. Overexpression of *OXTR* in adenomyosis-surrounding myometrium coupled with vasopressin receptor (VP1αR) expression in blood vessels and myometrium may contribute to altered microcirculation as well as increased uterine contractility (Mechsner et al., 2010). Collagen degradation and inflammation in the endometrium may also be involved in endometrium dysfunction and further HMB in adenomyosis with molecular mechanisms awaiting further definition.

### Strengths and challenges

The strength of the present study is that, to the best of our knowledge, it is the first comparison of endometrium and myometrium of women with and without diffuse adenomyosis at the transcriptomic level and subsequent analyses of biological processes and signalling pathways. Moreover, all specimens were obtained in one phase of the menstrual cycle (proliferative phase), avoiding different hormonal milieu across the cycle confounding data and interpretation. Although the results indicate that ECM remodelling in myometrium is involved in the pathogenesis of adenomyosis, ECM degradation and abnormal expression of MMPs have also been detected in leiomyomas (Islam et al., 2018). Since the probability of co-occurrence between adenomyosis and uterine fibroids is up to 70% (Upson et al., 2020), it is difficult to find cases without uterine fibroids. Although the location of fibroids by IHC was avoided when selecting uterine tissue for RNA-sequencing in both cases and controls, the coexistence of uterine fibroids in participants and controls recruited for this study is still considered a limitation of our study. Moreover, our result still needs to be validated in a larger sample size and future in-vivo animal models

In conclusion, our results support abnormalities in endometrium and myometrium of women who have adenomyosis compared with controls. The data strongly support the collective endometrial cell migration theory in the pathogenesis of adenomyosis, wherein inflammation induces ECM remodelling, creating a track for subsequent collective cell migration and onset of adenomyosis in the myometrium. Also, our results underscore the importance of PRL signalling in the endometrium and myometrium of women with versus without adenomyosis, providing opportunity for developing targeted treatments for the disease. Moreover, abnormal myometrial GABA synaptic transmission in the myometrium of women with disease also offers a novel target for innovation in management of dysmenorrhoea and chronic pelvic pain in women with adenomyosis.

## Supplementary Material

Suppl Table 1

Syppl Table 2

## Figures and Tables

**TABLE 1:** THE ENDOMETRIAL DIFFERENTIALLY EXPRESSED GENES THAT MAY CONTRIBUTE TO THE ABNORMAL COLLECTIVE CELL MIGRATION IN WOMEN WITH ADENOMYOSIS

Gene symbol	Log2FC	*P*-value	Biological processes
*MYO3A*	2.13597894	0.025098694	Cell–cell cohesion and coupling
*GJB2*	2.06355172	0.04771312	Cell–cell cohesion and coupling (gap junction)
*GJB3*	2.824542409	0.004018079	Cell–cell cohesion and coupling (gap junction)
*GJB4*	3.739644004	0.00401793	Cell–cell cohesion and coupling (gap junction)
*GJB5*	6.246065184	0.002934825	Cell–cell cohesion and coupling (gap junction)
*ITGB6*	2.663983313	0.002239706	Cell–cell cohesion and coupling (integrin)
*ICAM1*	4.013325458	3.38E-07	Cell–cell cohesion and coupling
*ICAM4*	2.487438086	0.017262748	Cell–cell cohesion and coupling
*CLDN14*	3.483301242	0.00244102	Cell–cell cohesion and coupling (tight junction)
*CLDN6*	3.037327159	0.010841124	Cell–cell cohesion and coupling (tight junction)
*CLDN8*	−2.527504706	0.037041349	Cell–cell cohesion and coupling (tight junction)
*MMP9*	3.84882174	1.98E-06	Forming a migration track, ECM remodelling
*MMP1*	8.041923495	1.07E-05	Forming a migration track, ECM remodelling
*MMP10*	5.1870582	0.002465975	Forming a migration track, ECM remodelling
*MMP12*	2.769143318	0.001400788	Forming a migration track, ECM remodelling
*MMP13*	4.002987481	0.00255607	Forming a migration track, ECM remodelling
*MMP27*	4.156066119	0.002233888	Forming a migration track, ECM remodelling
*MMP3*	6.455516004	0.000462347	Forming a migration track, ECM remodelling
*MMP7*	2.279017256	0.019064915	Forming a migration track, ECM remodelling
*MMP8*	10.39749709	0.000220408	Forming a migration track, ECM remodelling
*HAS1*	2.77613436	0.02534036	Forming a migration track, ECM remodelling
*HAS2*	2.85980488	0.00077117	Forming a migration track, ECM remodelling
*HAS3*	2.73840812	1.92E-05	Forming a migration track, ECM remodelling
*LAMB3*	2.899319784	0.008113949	Forming a migration track, ECM remodelling
*COL28A1*	−2.049272211	0.002893567	Forming a migration track, ECM remodelling
*CXCL1*	3.437751705	0.001022512	Driving force of guided cell migration
*CXCL10*	2.466611485	0.00463707	Driving force of guided cell migration
*CXCL13*	−5.778651129	2.00E-05	Driving force of guided cell migration
*CXCL14*	−3.86665238	0.000483943	Driving force of guided cell migration
*CXCL2*	3.427204022	0.00020155	Driving force of guided cell migration
*CXCL3*	3.714065968	6.73E-06	Driving force of guided cell migration
*CXCL5*	5.128974018	3.87E-06	Driving force of guided cell migration
*CXCL6*	3.676691233	0.001805715	Driving force of guided cell migration
*CXCL8*	4.550862093	8.02E-06	Driving force of guided cell migration

ECM, extracellular matrix.

**TABLE 2:** THE ENRICHMENT OF COMMON DIFFERENTIALLY EXPRESSED GENES IN ENDOMETRIUM AND MYOMETRIUM

Biological processes	Genes
GO:0030574~collagen catabolic process	*MMP13, MMP27, MMP1, MMP3, MMP8*
GO:0022617~extracellular matrix disassembly	*MMP13, MMP1, MMP3, TPSAB1, MMP8*
GO:0006508~proteolysis	*TPSB2, MMP13, MMP27, MMP1, MMP3, TPSAB1, MMP8*
GO:0006955~immune response	*CXCL6, CSF3, CXCL8, CXCL13, CXCL14, IL2*
GO:0006954~inflammatory response	*CXCL6, SERPINA3, CXCL8, KRT16, CXCL13*
GO:0006935~chemotaxis	*CXCL6, CXCL8, CXCL14*
GO:0007165~signal transduction	*CHRNA1, CXCL6, CXCL8, SOX9, CXCL14*
GO:0008284~positive regulation of cell proliferation	*CSF3, BNC1, SOX9, IL2, EREG*
GO:0045944~positive regulation of transcription from RNA polymerase II promoter	*FOXA1, CSF3, ELF5, FOXH1, WNT7A, SOX9, PITX1, FOXA3, IL2*
GO:0030154~cell differentiation	*BNC1, ELF5, MAL, FOXA3*
KEGG path hsa04060: cytokine–cytokine receptor interaction	*CXCL6, CSF3, CXCL8, CXCL13, CXCL14, IL2*
KEGG path hsa04917: prolactin signalling pathway	*SHC4, CCND1, GALT, SOCS5, ELF5*
KEGG path hsa04062: chemokine signalling pathway	*CXCL6, CXCL8, CXCL13, CXCL14*

KEGG, Kyoto Encyclopedia of Genes and Genomes.

## KEY MESSAGE

This study supports invasive endometrium theory in the pathogenesis of adenomyosis, proving evidence for the vital role of endometrial collective cell migration. Abnormal myometrial GABA synaptic transmission and prolactin signalling abnormalities may contribute to dysmenorrhoea in women with adenomyosis and as a possible target for novel therapeutic development.

## References

[R1] Ahmed SBM, Prigent SA Insights into the Shc Family of Adaptor Proteins. J. Mol. Signal 2017; 12: 2 30210578 10.5334/1750-2187-12-2 PMC5624076

[R2] Benagiano G, Habiba M, Brosens I The pathophysiology of uterine adenomyosis: an update. Fertil. Steril. 2012; 98: 572–579 22819188 10.1016/j.fertnstert.2012.06.044

[R3] Carrarelli P, Luddi A, Funghi L, Arcuri F, Batteux F, Dela Cruz C, Tosti C, Reis FM, Chapron C, Petraglia F Urocortin and corticotrophin-releasing hormone receptor type 2 mRNA are highly expressed in deep infiltrating endometriotic lesions. Reprod. Biomed Online 2016; 33: 476–483 10.1016/j.rbmo.2016.07.009 27567427

[R4] Chun S, Kim YM, Ji YI Uterine adenomyosis which developed from hypoplastic uterus in postmenopausal woman with mayer-rokitansky-kuster-hauser syndrome: a case report. J. Menopausal. Med. 2013; 19: 135–138 25371879 10.6118/jmm.2013.19.3.135 PMC4217560

[R5] Devi YS, Shehu A, Halperin J, Stocco C, Le J, Seibold AM, Gibori G Prolactin signaling through the short isoform of the mouse prolactin receptor regulates DNA binding of specific transcription factors, often with opposite effects in different reproductive issues. Reprod. Biol. Endocrinol. 2009; 7: 87 19703295 10.1186/1477-7827-7-87 PMC2746216

[R6] Ding K, Yuan Y, Chong QY, Yang Y, Li R, Li X, Kong X, Qian P, Xiong Z, Pandey V, Ma L, Wu Z, Lobie PE, Zhu T Autocrine Prolactin Stimulates Endometrial Carcinoma Growth and Metastasis and Reduces Sensitivity to Chemotherapy. Endocrinology 2017; 158: 1595–1611 28204229 10.1210/en.2016-1903

[R7] Dobin A, Davis CA, Schlesinger F, Drenkow J, Zaleski C, Jha S, Batut P, Chaisson M, Gingeras TR STAR: ultrafast universal RNA-seq aligner. Bioinformatics 2013; 29: 15–21 23104886 10.1093/bioinformatics/bts635 PMC3530905

[R8] Donnez O, Orellana R, Van Kerk O, Dehoux JP, Donnez J, Dolmans MM Invasion process of induced deep nodular endometriosis in an experimental baboon model: similarities with collective cell migration? Fertil. Steril. 2015; 104 10.1016/j.fertnstert.2015.05.011 26049053

[R9] Friedl P, Gilmour D Collective cell migration in morphogenesis, regeneration and cancer. Nat. Rev. Mol. Cell Biol. 2009; 10: 445–457 19546857 10.1038/nrm2720

[R10] Friedl P, Hegerfeldt Y, Tusch M Collective cell migration in morphogenesis and cancer. Int. J. Dev. Biol. 2004; 48: 441–449 15349818 10.1387/ijdb.041821pf

[R11] Garcia-Solares J, Donnez J, Donnez O, Dolmans MM Pathogenesis of uterine adenomyosis: invagination or metaplasia? Fertil. Steril. 2018; 109: 371–379 29566849 10.1016/j.fertnstert.2017.12.030

[R12] Goumenou AG, Arvanitis DA, Matalliotakis IM, Koumantakis EE, Spandidos DA Loss of heterozygosity in adenomyosis on hMSH2, hMLH1, p16Ink4 and GALT loci. Int. J. Mol. Med. 2000; 6: 667–671 11078826 10.3892/ijmm.6.6.667

[R13] Greenfield LJ Jr., Zaman SH, Sutherland ML, Lummis SC, Niemeyer MI, Barnard EA, Macdonald RL Mutation of the GABAA receptor M1 transmembrane proline increases GABA affinity and reduces barbiturate enhancement. Neuropharmacology 2002; 42: 502–521 11955521 10.1016/s0028-3908(01)00196-4

[R14] Guo SW, Mao X, Ma Q, Liu X Dysmenorrhea and its severity are associated with increased uterine contractility and overexpression of oxytocin receptor (OTR) in women with symptomatic adenomyosis. Fertil. Steril. 2013; 99: 231–240 22999795 10.1016/j.fertnstert.2012.08.038

[R15] Harada T, Khine YM, Kaponis A, Nikellis T, Decavalas G, Taniguchi F The Impact of Adenomyosis on Women’s Fertility. Obstet. Gynecol. Surv. 2016; 71: 557–568 27640610 10.1097/OGX.0000000000000346 PMC5049976

[R16] Harmsen MJ, Wong CFC, Mijatovic V, Griffioen AW, Groenman F, Hehenkamp WJK, Huirne JAF Role of angiogenesis in adenomyosis-associated abnormal uterine bleeding and subfertility: a systematic review. Hum. Reprod Update 2019; 25: 647–671 10.1093/humupd/dmz024 PMC6737562 31504506

[R17] Hoo PS, Norhaslinda AR, Reza JN Rare Case of Leiomyoma and Adenomyosis in Mayer-Rokitansky-Kuster-Hauser Syndrome. Case Rep. Obstet. Gynecol. 2016; 20163725043 10.1155/2016/3725043 PMC5097798 27843659

[R18] Islam MS, Ciavattini A, Petraglia F, Castellucci M, Ciarmela P Extracellular matrix in uterine leiomyoma pathogenesis: a potential target for future therapeutics. Hum. Reprod Update 2018; 24: 59–85 10.1093/humupd/dmx032 29186429

[R19] Jiang D, Christ S, Correa-Gallegos D, Ramesh P, Kalgudde Gopal S, Wannemacher J, Mayr CH, Lupperger V, Yu Q, Ye H, Muck-Hausl M, Rajendran V, Wan L, Liu J, Mirastschijski U, Volz T, Marr C, Schiller HB, Rinkevich Y Injury triggers fascia fibroblast collective cell migration to drive scar formation through N-cadherin. Nat. Commun. 2020; 11: 5653 33159076 10.1038/s41467-020-19425-1 PMC7648088

[R20] Jiang Y, Jiang R, Cheng X, Zhang Q, Hu Y, Zhang H, Cao Y, Zhang M, Wang J, Ding L, Diao Z, Sun H, Yan G Decreased expression of NR4A nuclear receptors in adenomyosis impairs endometrial decidualization. Mol. Hum. Reprod. 2016; 22: 655–668 27515096 10.1093/molehr/gaw042

[R21] Kissler S, Zangos S, Kohl J, Wiegratz I, Rody A, Gatje R, Vogl TJ, Kunz G, Leyendecker G, Kaufmann M Duration of dysmenorrhoea and extent of adenomyosis visualised by magnetic resonance imaging. Eur. J. Obstet. Gynecol. Reprod. Biol. 2008; 137: 204–209 17397990 10.1016/j.ejogrb.2007.01.015

[R22] Klohonatz KM, Coleman SJ, Islas-Trejo AD, Medrano JF, Hess AM, Kalbfleisch T, Thomas MG, Bouma GJ, Bruemmer JE Coding RNA Sequencing of Equine Endometrium during Maternal Recognition of Pregnancy. Genes (Basel) 2019: 10 10.3390/genes10100749 PMC6826732 31557877

[R23] Lee JG, Heur M Interleukin-1beta enhances cell migration through AP-1 and NF-kappaB pathway-dependent FGF2 expression in human corneal endothelial cells. Biol. Cell 2013; 105: 175–189 23331079 10.1111/boc.201200077 PMC3618530

[R24] Leyendecker G, Wildt L, Mall G The pathophysiology of endometriosis and adenomyosis: tissue injury and repair. Arch. Gynecol. Obstet. 2009; 280: 529–538 19644696 10.1007/s00404-009-1191-0 PMC2730449

[R25] Li S, Zhai J, Liu J, Di F, Sun Y, Li W, Chen ZJ, Du Y Erythropoietin-producing hepatocellular A7 triggering ovulation indicates a potential beneficial role for polycystic ovary syndrome. EBioMedicine 2018; 36: 539–552 30292674 10.1016/j.ebiom.2018.09.046 PMC6197718

[R26] Liu Z, Sun Z, Liu H, Niu W, Wang X, Liang N, Wang X, Wang Y, Shi Y, Xu L, Shi W Single-cell transcriptomic analysis of eutopic endometrium and ectopic lesions of adenomyosis. Cell Biosci. 2021; 11: 51 33685511 10.1186/s13578-021-00562-z PMC7938473

[R27] Mechsner S, Grum B, Gericke C, Loddenkemper C, Dudenhausen JW, Ebert AD Possible roles of oxytocin receptor and vasopressin-1alpha receptor in the pathomechanism of dysperistalsis and dysmenorrhea in patients with adenomyosis uteri. Fertil. Steril. 2010; 94: 2541–2546 20413116 10.1016/j.fertnstert.2010.03.015

[R28] Montell DJ Morphogenetic cell movements: diversity from modular mechanical properties. Science 2008; 322: 1502–1505 19056976 10.1126/science.1164073

[R29] Mori T, Nagasawa H, Takahashi S The induction of adenomyosis in mice by intrauterine pituitary isografts. Life Sci. 1981; 29: 1277–1282 7300555 10.1016/0024-3205(81)90234-4

[R30] Nie J, Liu X, Guo SW Immunoreactivity of oxytocin receptor and transient receptor potential vanilloid type 1 and its correlation with dysmenorrhea in adenomyosis. Am. J. Obstet. Gynecol 2010; 202 10.1016/j.ajog.2009.11.035 20096818

[R31] Noyes RW, Hertig AT, Rock J Dating the endometrial biopsy. Am. J. Obstet. Gynecol. 1975; 122: 262–263 1155504 10.1016/s0002-9378(16)33500-1

[R32] Sengupta P, Sharma A, Mazumdar G, Banerjee I, Tripathi SK, Bagchi C, Das N The possible role of fluoxetine in adenomyosis: an animal experiment with clinical correlations. J. Clin. Diagn. Res. 2013; 7: 1530–1534 23998115 10.7860/JCDR/2013/5654.3128 PMC3749685

[R33] Singtripop T, Mori T, Park MK, Sakamoto S, Kawashima S Development of uterine adenomyosis after treatment with dopamine antagonists in mice. Life Sci. 1991; 49: 201–206 2062175 10.1016/0024-3205(91)90004-u

[R34] Strieter RM, Burdick MD, Mestas J, Gomperts B, Keane MP, Belperio JA Cancer CXC chemokine networks and tumour angiogenesis. Eur. J. Cancer 2006; 42: 768–778 16510280 10.1016/j.ejca.2006.01.006

[R35] Thuroff F, Goychuk A, Reiter M, Frey E Bridging the gap between single-cell migration and collective dynamics. Elife 2019; 8 10.7554/eLife.46842 PMC6992385 31808744

[R36] Upson K, Missmer SA Epidemiology of Adenomyosis. Semin. Reprod. Med. 2020; 38: 89–107 33105509 10.1055/s-0040-1718920 PMC7927213

[R37] Vannuccini S, Tosti C, Carmona F, Huang SJ, Chapron C, Guo SW, Petraglia F Pathogenesis of adenomyosis: an update on molecular mechanisms. Reprod. Biomed. Online 2017; 35: 592–601 28693952 10.1016/j.rbmo.2017.06.016

[R38] Wang J, Movilla P, Chen T, Wang J, Morales B, Williams A, Reddy H, Tavcar J, Morris S, Loring M, Isaacson K Concomitant Adenomyosis among Patients with Asherman Syndrome. J. Minim. Invasive Gynecol. 2021; 28 10.1016/j.jmig.2020.07.011 32712321

[R39] Wang T, Hamilla S, Cam M, Aranda-Espinoza H, Mili S Extracellular matrix stiffness and cell contractility control RNA localization to promote cell migration. Nat. Commun. 2017; 8: 896 29026081 10.1038/s41467-017-00884-y PMC5638855

[R40] Wara-aswapati N, Surarit R, Chayasadom A, Boch JA, Pitiphat W RANKL upregulation associated with periodontitis and Porphyromonas gingivalis. J. Periodontol. 2007; 78: 1062–1069 17539720 10.1902/jop.2007.060398

[R41] Ward CM, To TH, Pederson SM ngsReports: a Bioconductor package for managing FastQC reports and other NGS related log files. Bioinformatics 2020; 36: 2587–2588 31841127 10.1093/bioinformatics/btz937

[R42] Xiang Y, Sun Y, Yang B, Yang Y, Zhang Y, Yu T, Huang H, Zhang J, Xu H Transcriptome sequencing of adenomyosis eutopic endometrium: A new insight into its pathophysiology. J. Cell. Mol. Med. 2019; 23: 8381–8391 31576674 10.1111/jcmm.14718 PMC6850960

[R43] Yam MF, Loh YC, Tan CS, Khadijah Adam S, Abdul Manan N, Basir R General Pathways of Pain Sensation and the Major Neurotransmitters Involved in Pain Regulation. Int. J. Mol. Sci. 2018; 19 10.3390/ijms19082164 PMC6121522 30042373

[R44] Yan D, Liu X, Guo SW Neuropeptides Substance P and Calcitonin Gene Related Peptide Accelerate the Development and Fibrogenesis of Endometriosis. Sci. Rep. 2019; 9: 2698 30804432 10.1038/s41598-019-39170-w PMC6389969

[R45] Zhai J, Vannuccini S, Petraglia F, Giudice LC Adenomyosis: Mechanisms and Pathogenesis. Semin. Reprod. Med. 2020; 38: 129–143 33032339 10.1055/s-0040-1716687 PMC7932680

[R46] Zhang M, Liu S, Chua MS, Li H, Luo D, Wang S, Zhang S, Han B, Sun C SOCS5 inhibition induces autophagy to impair metastasis in hepatocellular carcinoma cells via the PI3K/Akt/mTOR pathway. Cell Death Dis. 2019; 10: 612 31406106 10.1038/s41419-019-1856-y PMC6690952

[R47] Zhang X, Lin J, Ma Y, Zhao J Overexpression of E74-Like Factor 5 (ELF5) Inhibits Migration and Invasion of Ovarian Cancer Cells. Med. Sci. Monit. 2019; 25: 856–865 30696803 10.12659/MSM.913058 PMC6364457

[R48] Zhou L, Guo X, Jing BA, Zhao L CD44 is involved in CXCL-12 induced acute myeloid leukemia HL-60 cell polarity. Biocell. 2010; 34: 91–94 20925198

