# Supplementary material for: Transcriptomic analysis supports collective endometrial cell migration in the pathogenesis of adenomyosis
Source: Reprod Biomed Online. Author manuscript; Available in PMC 2023 Mar 1. (PMC9976941; doi:10.1016/j.rbmo.2022.05.007)
Supplement: Suppl Table 1 [file NIHMS1870832-supplement-Suppl_Table_1.docx]

**Supplementary Table 1. Demographic characteristics of recruited participants with or without adenomyosis.**

|  | **Women without Adenomyosis** | | **Women with Adenomyosis** |
| --- | --- | --- | --- |
| Age (years) | 40.00 ± 5.28 | 43.06 ± 3.13 | |
| Diagnosis | Leiomyoma (N=10)  Pelvic pain (N=2)  Menorrhagia (N=3) | Adenomyosis (N=6)  Endometriosis and Adenomyosis (N=3)  Adenomyosis and Leiomyoma (N=7) | |
| Endometrial Phase | Proliferative phase | Proliferative phase | |
