# Supplementary material for: Transcriptomic analysis supports collective endometrial cell migration in the pathogenesis of adenomyosis
Source: Reprod Biomed Online. Author manuscript; Available in PMC 2023 Mar 1. (PMC9976941; doi:10.1016/j.rbmo.2022.05.007)
Supplement: Syppl Table 2 [file NIHMS1870832-supplement-Syppl_Table_2.docx]

**Supplementary Table 2. Primer sequence of target genes**

| **Gene symbol** | **Primer sequence** |
| --- | --- |
| IL1B (forward) | 5’-TTCGACACATGGGATAACGAGG-3’ |
| IL1B (reverse) | 5’-TTTTTGCTGTGAGTCCCGGAG -3’ |
| IL18 (forward) | 5’-TCTTCATTGACCAAGGAAATCGG-3’ |
| IL18 (reverse) | 5’-TCCGGGGTGCATTATCTCTAC -3’ |
| TNF (forward) | 5’-GAGGCCAAGCCCTGGTATG-3’ |
| TNF (reverse) | 5’-CGGGCCGATTGATCTCAGC -3’ |
| MMP1 (forward) | 5’-AAAATTACACGCCAGATTTGCC-3’ |
| MMP1 (reverse) | 5’-GGTGTGACATTACTCCAGAGTTG -3’ |
| MMP8 (forward) | 5’-TGCTCTTACTCCATGTGCAGA-3’ |
| MMP8 (reverse) | 5’-TCCAGGTAGTCCTGAACAGTTT -3’ |
| MMP13 (forward) | 5’-ACTGAGAGGCTCCGAGAAATG -3’ |
| MMP13 (reverse) | 5’-GAACCCCGCATCTTGGCTT -3’ |
| TIMP1 (forward) | 5’-CTTCTGCAATTCCGACCTCGT-3’ |
| TIMP1 (reverse) | 5’-ACGCTGGTATAAGGTGGTCTG -3’ |
| COL1A1 (forward) | 5’-GAGGGCCAAGACGAAGACATC-3’ |
| COL1A1 (reverse) | 5’-CAGATCACGTCATCGCACAAC -3’ |
| COL1A2 (forward) | 5’-GAGCGGTAACAAGGGTGAGC-3’ |
| COL1A2 (reverse) | 5’-CTTCCCCATTAGGGCCTCTC -3’ |
| COL3A1 (forward) | 5’-TTGAAGGAGGATGTTCCCATCT-3’ |
| COL3A1 (reverse) | 5’-ACAGACACATATTTGGCATGGTT -3’ |
| HAS1 (forward) | 5’-GAGCCTCTTCGCGTACCTG-3’ |
| HAS1 (reverse) | 5’-CCTCCTGGTAGGCGGAGAT -3’ |
| HAS2 (forward) | 5’-CTCTTTTGGACTGTATGGTGCC-3’ |
| HAS2 (reverse) | 5’-AGGGTAGGTTAGCCTTTTCACA -3’ |
| HAS3 (forward) | 5’-CAGCCTATGTGACGGGCTAC-3’ |
| HAS3 (reverse) | 5’-CCTCCTGGTATGCGGCAAT -3’ |
| CD44 (forward) | 5’-CTGCCGCTTTGCAGGTGTA-3’ |
| CD44 (reverse) | 5’-CATTGTGGGCAAGGTGCTATT-3’ |
| CXCL8 (forward) | 5’- ACTGAGAGTGATTGAGAGTGGAC-3’ |
| CXCL8 (reverse) | 5’- AACCCTCTGCACCCAGTTTTC -3’ |
| GABRA2 (forward) | 5’-GCTGGCTAACATCCAAGAAGAT-3’ |
| GABRA2 (reverse) | 5’-GCCGATTATCGTAACCATCCAGA -3’ |
| NTS (forward) | 5’-TGCTTTAGATGGCTTTAGCTTGG-3’ |
| NTS (reverse) | 5’-TTCCTGGATTAACTCCCAGTGT -3’ |
| OXTR (forward) | 5’-AAGCCTCGGCCTTCATCATC-3’ |
| OXTR (reverse) | 5’-TTTTGCTGGCACTCGTCTCT -3’ |
| CCND1 (forward) | 5’-GCTGCGAAGTGGAAACCAT-3’ |
| CCND1 (reverse) | 5’-CCTCCTTCTGCACACATTTGAA-3’ |
| GALT (forward) | 5’-CGCAGTGGAACCGATCCTC-3’ |
| GALT (reverse) | 5’-GATGGTCGTTTGCCCGGAA-3’ |
| β-ACTIN (forward) | 5′-GGGAAATCGTGCGTGACATTAAG-3′ |
| β-ACTIN (reverse) | 5′-TGTGTTGGCGTACAGGTCTTTG-3′ |
